# Global landscape of COVID-19 research: a visualization analysis of randomized clinical trials

**DOI:** 10.1007/s10238-023-01254-3

**Published:** 2024-01-22

**Authors:** Sa’ed H. Zyoud

**Affiliations:** 1 Department of Clinical and Community Pharmacy, College of Medicine and Health Sciences, An-Najah National University, Nablus, 44839 Palestine; 2 Clinical Research Centre, An-Najah National University Hospital, Nablus, 44839 Palestine

**Keywords:** Bibliometric, COVID-19, Visualization, VOSviewer, Scopus, Randomized controlled trials

## Abstract

The emergence of COVID-19 in 2019 has resulted in a significant global health crisis. Consequently, extensive research was published to understand and mitigate the disease. In particular, randomized controlled trials (RCTs) have been considered the benchmark for assessing the efficacy and safety of interventions. Hence, the present study strives to present a comprehensive overview of the global research landscape pertaining to RCTs and COVID-19. A bibliometric analysis was performed using the Scopus database. The search parameters included articles published from 2020 to 2022 using keywords specifically related to COVID-19 and RCTs. The data were analyzed using various bibliometric indicators. The volume of publications, contributions of countries and institutions, funding agencies, active journals, citation analysis, co-occurrence analysis, and future research direction analysis were specifically analyzed. A total of 223,480 research articles concerning COVID-19 were published, with 3,727 of them related to RCTs and COVID-19. The ten most productive countries collectively produced 75.8% of the documents, with the United States leading the way by contributing 31.77%, followed by the UK with 14.03% (*n* = 523), China with 12.96% (*n* = 483) and Canada with 7.16% (*n* = 267). *Trials* (*n* = 173, 4.64%), *BMJ Open* (*n* = 81, 2.17%), *PLOS One* (*n* = 73, 1.96%) and *JAMA Network Open* (*n* = 53, 1.42%) were the most active journals in publishing articles related to COVID-19 RCTs. The co-occurrence analysis identified four clusters of research areas: the safety and effectiveness of COVID-19 vaccines, mental health strategies to cope with the impact of the pandemic, the use of monoclonal antibodies to treat patients with COVID-19, and systematic reviews and meta-analyses of COVID-19 research. This paper offers a detailed examination of the global research environment pertaining to RCTs and their use in the context of the COVID-19 pandemic. The comprehensive body of research findings was found to have been generated by the collaborative efforts of multiple countries, institutions, and funding organizations. The predominant research areas encompassed COVID-19 vaccines, strategies for mental health, monoclonal antibodies, and systematic reviews. This information has the potential to aid researchers, policymakers, and funders in discerning areas of weakness and establishing areas of priority.

## Background

The COVID-19 pandemic, which emerged in 2019, has rapidly expanded throughout numerous countries worldwide, resulting in a substantial public health crisis with effects at an international level [1]. On May 3, 2023, the epidemic had a significant impact, resulting in over 765 million confirmed cases and a mortality rate of 6.92 million [2].

The COVID-19 pandemic has prompted researchers, regulators, and policymakers to respond promptly by finding strategies for preventing the pandemic [3]. Nevertheless, the scientific community has faced challenges in generating dependable data to inform and direct these strategies. The delays experienced can be attributed, at least in part, to the intricacies involved in conducting clinical trials amidst a pandemic. These complexities encompass challenges related to participant recruitment and the assurance of their safety [4]. Furthermore, the global pandemic has exerted immense pressure on healthcare systems and economics, thereby impeding the progress of developing and evaluating novel medical interventions. Given the challenges mentioned above, the Food and Drug Administration (FDA) has formulated regulatory guidelines for implementing clinical studies in the context of pandemics [5]. Notably, regulatory measures have exhibited more flexibility throughout the current period. However, achieving an appropriate balance in resource allocation, including financial and human resources, is crucial for studies pertaining to COVID-19 and non-COVID-19 subjects.

The COVID-19 pandemic has highlighted the need for well-designed randomized clinical trials (RCTs). Several studies have shown uncertain outcomes, and most COVID-19 clinical trials have been undertaken without adequate methods or planning [4, 6–9]. These trials evaluate herbal preparations, invasive medical procedures, vaccines, and experimental stem cell therapy in patients ranging from pre-exposure prophylaxis to critically ill hospitalized patients. Few trials have examined pre- or post-exposure to COVID-19, and most have focused on hospitalized patients [10–13]. More than 100 therapeutic compounds are being studied, although many are repurposed medications such as hydroxychloroquine and lopinavir-ritonavir for COVID-19. Most of these studies typically enroll fewer than 100 participants for experimental intervention, resulting in overlap and duplication [4, 9, 14].

The global impact of the COVID-19 pandemic has been significant, highlighting the crucial necessity for new vaccines and treatments [15]. Despite the lack of known therapy, researchers and medical professionals have worked tirelessly to conduct clinical trials and discover novel treatments for the disease [16, 17]. As evident from the vast array of medical research, with more than 300,000 articles currently available in PubMed, the epidemic has spurred a wealth of investigations on various aspects of the virus [18].

Randomized clinical trials are crucial in advancing the understanding and development of COVID-19 treatments. These trials offer a rigorous and controlled approach to evaluating the safety and effectiveness of interventions [19, 20], making them the most reliable method for evaluating health interventions. The design of RCTs is structured to minimize bias, which is why their results are considered the gold standard in medical research, and their impact on medical care is unmatched compared to other study designs [21]. As evidence-based medicine gains traction, the number and scope of RCTs are increasing exponentially, expanding their utility beyond their original purpose of testing pharmaceutical drugs, including evaluating treatments, devices, and diagnostics.

Despite the vast body of COVID-19 research, a comprehensive understanding of the global research landscape requires a comprehensive analysis of RCTs. A comprehensive summary of the current status of COVID-19 RCTs is not yet available, although numerous studies have focused on specific therapies. Consequently, the purpose of this study is to provide a comprehensive outline of COVID-19 investigation by conducting a review of the literature, identifying potential research gaps, and highlighting the most crucial topics. The purpose of this study is to enhance our understanding of the global research landscape pertaining to COVID-19 through a thorough examination of COVID-19 RCTs using literature analysis and visual aids.

Bibliometric analysis has gained widespread acceptance as a quantitative and qualitative method for evaluating global research contributions within a particular literary field [22–24]. Bibliometric research has garnered significant interest due to its efficacy in forecasting research trends in both established and emerging fields [25, 26]. This particular analytic method has been extensively employed across several academic fields [27–34], highlighting its effectiveness in supporting the process of decision-making. In recent years, there has been a notable surge in scholarly investigations focused on scientometric and bibliometric analyses pertaining to the COVID-19 pandemic [35–40]. The primary outcome of this study is to facilitate evidence-based decision-making among academics, policymakers, and medical practitioners by identifying potential areas of research advancement and alternative treatment options within the domain of COVID-19. This work has the potential to make a significant contribution to worldwide endeavors aimed at minimizing the impact of the COVID-19 pandemic.

## Methods

### Study design

Using bibliometric tools, a descriptive cross-sectional study was conducted on publications related to COVID-19 RCTs.

### Data source

Scopus is a well-known abstracting and citation database owned by Elsevier and used to search for published works. It features advanced scientific search engines and databases to obtain library data, making it a popular choice among academics. Unlike databases such as Web of Science, PubMed, and Google Scholar, Scopus is widely acknowledged as the leading quality-oriented database globally. It offers a more extensive and standardized repository of scientific literature, facilitating access to research work in diverse fields of inquiry [41, 42]. However, despite commendable endeavors by both WoS and Scopus to extend their inclusiveness, particularly in the last decade, the same applies to incorporating non-English language materials and regionally significant sources. Consequently, the primary biases toward an overabundance of English-language content, an uneven portrayal of nations, and an insufficient representation of literature in the social sciences and humanities persist as key constraints within these data repositories. However, numerous investigations have shown that Scopus delivers more extensive coverage in terms of both publications and citations in diverse fields and document categories. Additionally, it presents a more comprehensive rendering of non-English and localized literature. As a result, Scopus emerges as a more favorable selection for undertaking endeavors in the sphere of arts and humanities, especially when engaging in inventive and domestically focused research. This is particularly relevant when evaluating the caliber of sources in these domains, as WoS lacks impact metrics for such materials [43–46].

The author performed a bibliometric analysis using the SciVerse Scopus database. This analysis differs from systematic and scoping reviews [47–50] because it uses a comprehensive database to retrieve, review and visualize data, including information on citations and research collaboration. Systematic reviews can be broadly characterized as a form of research synthesis undertaken by specialized review groups. These groups aim to locate and retrieve global evidence relevant to specific questions [51–54]. They then assess and amalgamate the results of this inquiry to guide practice, policy, and, in some instances, future investigations [55]. In contrast, scoping reviews present an optimal approach to determine the extent and inclusiveness of a body of literature focused on a designated subject. They distinctly indicate the quantity of available literature and studies and provide a comprehensive (either broad or detailed) overview of their subject matter [48, 56–58]. Scoping reviews play a pivotal role in pinpointing voids within the literature, discerning the need for further research, and initially outlining the contours of the topic. They are particularly valuable for examining emerging evidence, especially when the potential for more specific questions in a more precise systematic review remains unclear. These reviews can delineate the types of evidence that address and influence practices within the field and the methodologies employed in the research [55].

### Search strategy

A detailed search was conducted using the Scopus database to gather pertinent information on COVID-19 RCTs. Publications issued from January 1, 2020, to December 31, 2022, had restrictions in the search conducted. To ensure accuracy and avoid any potential biases that may have been caused by constant updates and changes to the database, all relevant articles were retrieved and exported within a single day, specifically on April 23, 2023. The approach to data collection was precise and methodical, and an advanced search technique was utilized, which allowed filtering through a vast amount of literature to extract only the most relevant studies on the subject matter. By incorporating various keywords and phrases related to COVID-19 RCTs, a comprehensive search strategy was developed. Data for this study were retrieved using the following strategy:

- *Step 1* To achieve the goals of the study, COVID-19-associated terminology was entered into the Scopus research engine. This terminology was derived from various sources, including PubMed Medical Subject Headings (MeSH) and previous systematic and meta-analyses related to COVID-19 [59–62], as well as bibliometric studies focusing on COVID-19 [63–68]. All selected terms were then placed in the "Article Title" section.
- *Step 2* Subsequently, the documents found in the first step were narrowed down to only include those with the phrase "randomized controlled trials" and related terms in their titles or abstracts. The relevant terms for the randomized controlled trials were obtained from PubMed Medical Subject Headings (MeSH), as well as previous systematic reviews and meta-analyses on COVID-19 that involved RCTs [69–72].
- *Step 3* The scope of the retrieved documents was restricted to include only primary research articles while ignoring other forms of text, such as editorials, letters, and proceedings.

### Validation of the search strategy

The precision of the search strategy and the absence of bias were confirmed by two volunteers (S. A. and A. A.) by reviewing the titles and abstracts of the top 200 cited articles in the retrieved dataset. The retrieved document list from Scopus was subjected to systematic random sampling, where 10% of the list was taken. Every 10th, 20th, 30th, 40th document, and so on up to the end of the retrieved document list, was scrutinized by evaluating their titles and abstracts to avoid false positive outcomes. The research strategy was continuously refined until a completely accurate set of randomly selected outcomes was obtained. The research productivity of 20 active authors in the field was examined to validate the absence of false negative or missing results. A Spearman correlation test was used to compare the results obtained from the research strategy and the authors. The study revealed a strong and significant correlation (*p* < 0.001; *r* = 0.958) between the two sets of results, indicating the high level of validity of the research strategy. Notably, Sweileh et al. previously employed this validation approach [73–75].

### Bibliometric indicators

This research involved the examination of several bibliometric indicators, namely, (1) the total count of published works; (2) the impact factors of the top ten journals that had the most substantial influence; (3) the ten most frequently cited articles; (4) the research output levels of the top ten countries; (5) the research output levels of the top ten institutes; (6) the top ten funding agencies that supported research activities; and (7) citation patterns and the *h*-index.

### Data analysis and visualization

The dataset resulting from the final search query was extracted from Scopus as a CSV file. This file was utilized for both basic bibliometric analysis and advanced mapping techniques. To accomplish this, the CSV file containing the entire literature corpus was uploaded to VOSviewer version 1.6.19, a specialized online program designed to create visual maps based on user-defined queries. The interpretation of maps generated by the program is dependent on various factors, such as node size, color, line thickness, and proximity to other nodes [76, 77]. For instance, the size in a term map is determined by the frequency of occurrence of that term. In contrast, nodes of similar color represent a cluster that signifies a particular topic or research theme. To determine the main themes in the literature corpus, the most common terms in titles/abstracts with at least 100 appearances per term were mapped. VOSviewer also provides an overlay visualization option highlighting the most recent terms in yellow. The overlay visualization was based on the occurrence and average publication scores of the terms per year. When mapping countries, node size is proportional to the number of publications with joint authorship. Therefore, a larger node size indicates a higher degree of international collaboration.

## Results

### Volume of publications

Throughout the data collection period from 2020 to 2022, 223,480 research articles concerning COVID-19 were published. When narrowing the search down to articles related to COVID-19 RCTs, Scopus was able to pinpoint 3727 relevant articles. Among these 3727 examined documents, 638 (17.12%) were published in 2020, 1358 (36.44%) in 2021, and 1731 (46.44%) in 2022.

### Active countries

**Fig. 1:**
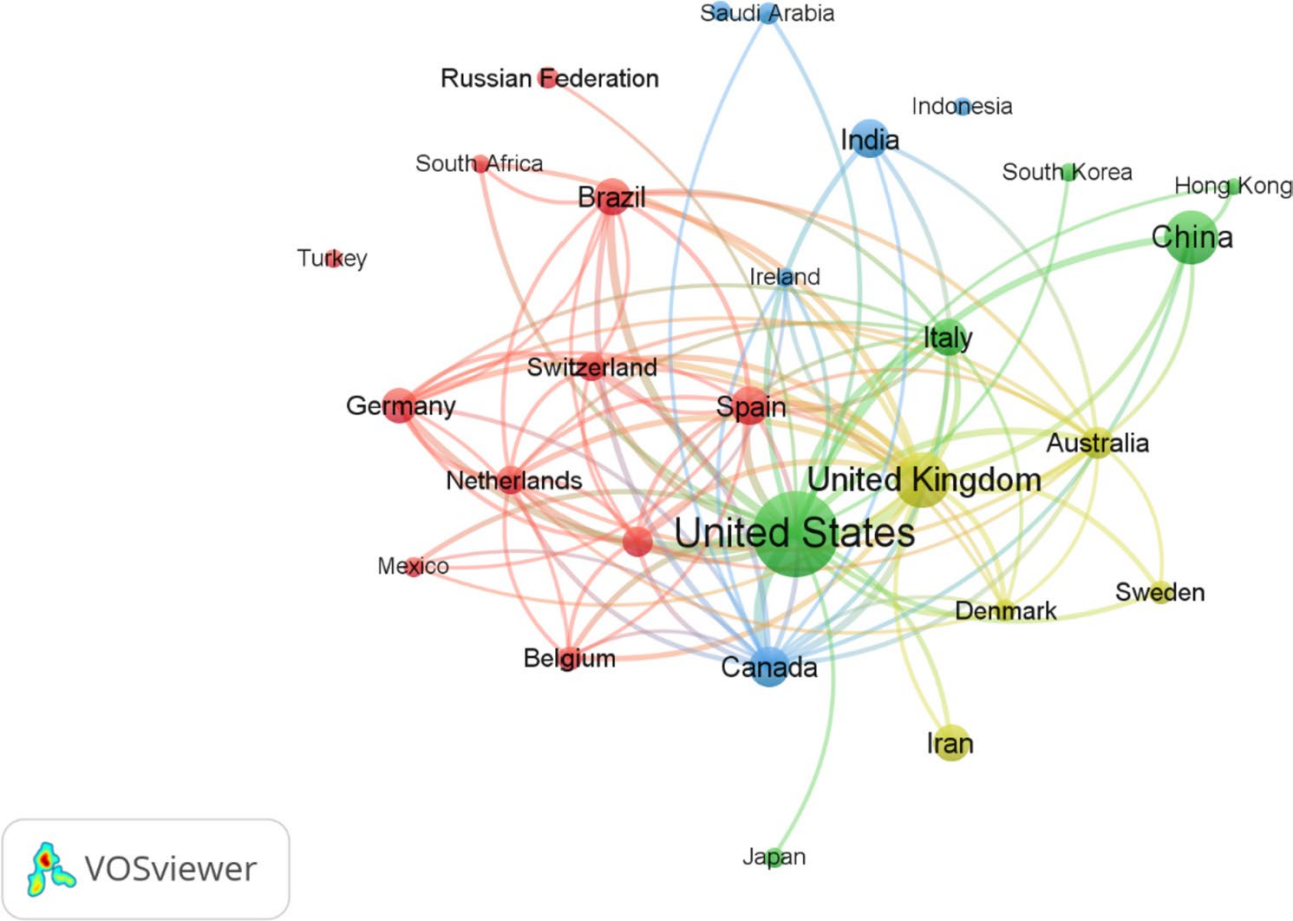
A network visualization map illustrating coauthorship collaborations among countries with more than 50 publications: of the 127 countries published in this field, 28 met the criterion. The size of the nodes on the map represents the number of publications by each country

The production of research related to COVID-19 RCTs was a collaborative effort among 127 countries. Table 1 highlights the top ten countries that were most productive in this regard. The analysis reveals that these ten countries collectively produced 75.8% of the documents, with the US leading the way by contributing 31.77%, followed by the UK with 14.03% (*n* = 523), China with 12.96% (*n* = 483) and Canada with 7.16% (*n* = 267). Furthermore, a network of countries was formed as depicted in Fig. 1, with each node representing a distinct country that produced at least 50 articles. The links between the nodes indicate collaborative relationships between countries. The USA and the UK emerged as the primary collaborators and have the strongest alliance ties in research with other countries.

**Table 1:** Publications related to COVID-19 RCTs from the ten most productive countries/regions

Ranking	Country	Number of documents	%
1st	United States	1184	31.77
2nd	United Kingdom	523	14.03
3rd	China	483	12.96
4th	Canada	267	7.16
5th	India	252	6.76
6th	Spain	247	6.63
7th	Iran	235	6.31
8th	Italy	232	6.22
9th	Brazil	222	5.96
10th	Germany	203	5.45

### Active institutions/organizations

Table 2 presents a list of the top ten institutions that have been actively involved in producing research publications on RCTs related to COVID-19. It is evident from the analysis that these institutions collectively contributed to 15.88% of the total documents published in this area. The leading position has been occupied by *Harvard Medical School*, which has produced 129 documents, accounting for 3.46% of the total publications. This is followed by the *University of Oxford* with 123 publications (3.30%), *Imperial College London* with 95 publications (2.55%), and the *University of Toronto* with 90 publications (2.41%). The majority of active institutions were based in the UK, with four institutions represented, followed by the United States with two institutions and one institution each from Canada, France, Brazil, and Iran.

**Table 2:** Top ten institutions publishing COVID-19 RCTs

Ranking	Institute	Country	*n*	%
1st	Harvard Medical School	USA	129	3.46
2nd	University of Oxford	UK	123	3.30
3rd	Imperial College London	UK	95	2.55
4th	University of Toronto	Canada	90	2.41
5th	INSERM	France	87	2.33
6th	Universidade de São Paulo	Brazil	80	2.15
7th	McMaster University	UK	79	2.12
8th	University College London	UK	78	2.09
9th	Brigham and Women's Hospital	USA	71	1.91
10th	Tehran University of Medical Sciences	Iran	63	1.69

### Analysis of funding agencies

Table 3 presents information on the top ten funding agencies that have actively engaged in producing articles related to COVID-19 RCTs. The data show that these funding agencies collectively contributed 17.54% of the documents. Among the leading contributors, the *National Institutes of Health* provided funding for the highest number of articles (*n* = 262; 7.03%), followed by the *National Natural Science Foundation of China* (*n* = 107; 2.87%) and the *National Institute of Allergy and Infectious Diseases* (*n* = 99; 2.66%), as indicated in Table 3.

**Table 3:** The top ten funding agencies with the most publications related to COVID-19 RCTs

Ranking	Funding agencies	Country	No. of publication	%
1st	National Institutes of Health	USA	262	7.03
2nd	National Natural Science Foundation of China	China	107	2.87
3rd	National Institute of Allergy and Infectious Diseases	USA	99	2.66
4th	National Heart, Lung, and Blood Institute	USA	88	2.36
5th	Pfizer	USA	83	2.23
6th	Gilead Sciences	USA	79	2.12
6th	National Institute for Health and Care Research	UK	79	2.12
8th	National Center for Advancing Translational Sciences	USA	74	1.99
9th	Medical Research Council	UK	68	1.82
10th	Wellcome Trust	UK	66	1.77

### Active journals

Table 4 shows details on the top ten journals that actively published articles related to COVID-19 RCTs. The data indicate that these funding agencies collectively contributed to 18.11% of the total published documents. Among the top ten journals, *trials (n* = *173,* 4.64%), *BMJ Open* (*n* = 81, 2.17%), *Plos One* (*n* = 73, 1.96%) and *JAMA Network Open* (*n* = 53, 1.42%) are the most prominent, as shown in Table 4.

**Table 4:** The top ten journals with the most publications related to COVID-19 RCTs

Ranking	Journal	No. of publication	%	IF^1^
1st	Trials	173	4.64	2.728
2nd	BMJ Open	81	2.17	3.006
3rd	Plos One	73	1.96	3.752
4th	JAMA Network Open	53	1.42	13.353
4th	New England Journal of Medicine	53	1.42	176.079
6th	Medicine	52	1.40	1.817
7th	International Journal of Environmental Research and Public Health	50	1.34	4.614
8th	Eclinicalmedicine	47	1.26	17.033
8th	Scientific Reports	47	1.26	4.996
10th	Lancet Respiratory Medicine	46	1.23	102.642

^1^Impact factor (IF) from Journal Citation Reports (Source Clarivate, 2022)

### Analysis of citations

Based on citation analysis, the average number of times the retrieved articles were cited was 42.47, and they achieved an h-index of 162 with a total of 157,211 citations. Citations for these articles ranged from 0 to 7517, with 561 of them not having any citations and 238 receiving 100 or more citations. The ten most-cited articles on COVID-19 RCTs received a total of 37,324 citations. The citations ranged from 1459 to 7517 [78–87]. This information can be found in Table 5.

**Table 5:** The top ten cited articles for publications related to COVID-19 RCTs

Ranking	Authors	Title	Year	Source title	Cited by
1st	Polack et al. [84]	“Safety and efficacy of the BNT162b2 mRNA Covid-19 vaccine”	2020	New England Journal of Medicine	7517
2nd	Horby et al. [83]	“Dexamethasone in hospitalized patients with covid-19”	2021	New England Journal of Medicine	5646
3rd	Baden et al. [78]	“Efficacy and safety of the mRNA-1273 SARS-CoV-2 vaccine”	2021	New England Journal of Medicine	5051
4th	Beigel et al. [79]	“Remdesivir for the treatment of COVID-19—Final report”	2020	New England Journal of Medicine	4160
5th	Cao et al. [80]	“A trial of lopinavir-ritonavir in adults hospitalized with severe covid-19”	2020	New England Journal of Medicine	3553
6th	Gautret et al. [82]	“Hydroxychloroquine and azithromycin as a treatment of COVID-19: results of an open-label non-randomized clinical trial”	2020	International Journal of Antimicrobial Agents	3452
7th	Voysey et al. [85]	“Safety and efficacy of the ChAdOx1 nCoV-19 vaccine (AZD1222) against SARS-CoV-2: an interim analysis of four randomised controlled trials in Brazil, South Africa, and the UK”	2021	The Lancet	2726
8th	Wang et al. [87]	“Remdesivir in adults with severe COVID-19: a randomised, double-blind, placebo-controlled, multicentre trial”	2020	The Lancet	2234
9th	Folegatti et al. [81]	“Safety and immunogenicity of the ChAdOx1 nCoV-19 vaccine against SARS-CoV-2: a preliminary report of a phase 1/2, single-blind, randomised controlled trial”	2020	The Lancet	1526
10th	Walsh et al. [86]	“Safety and immunogenicity of two RNA-based covid-19 vaccine candidates”	2020	New England Journal of Medicine	1459

### Co-occurrence analysis

**Fig. 2:**
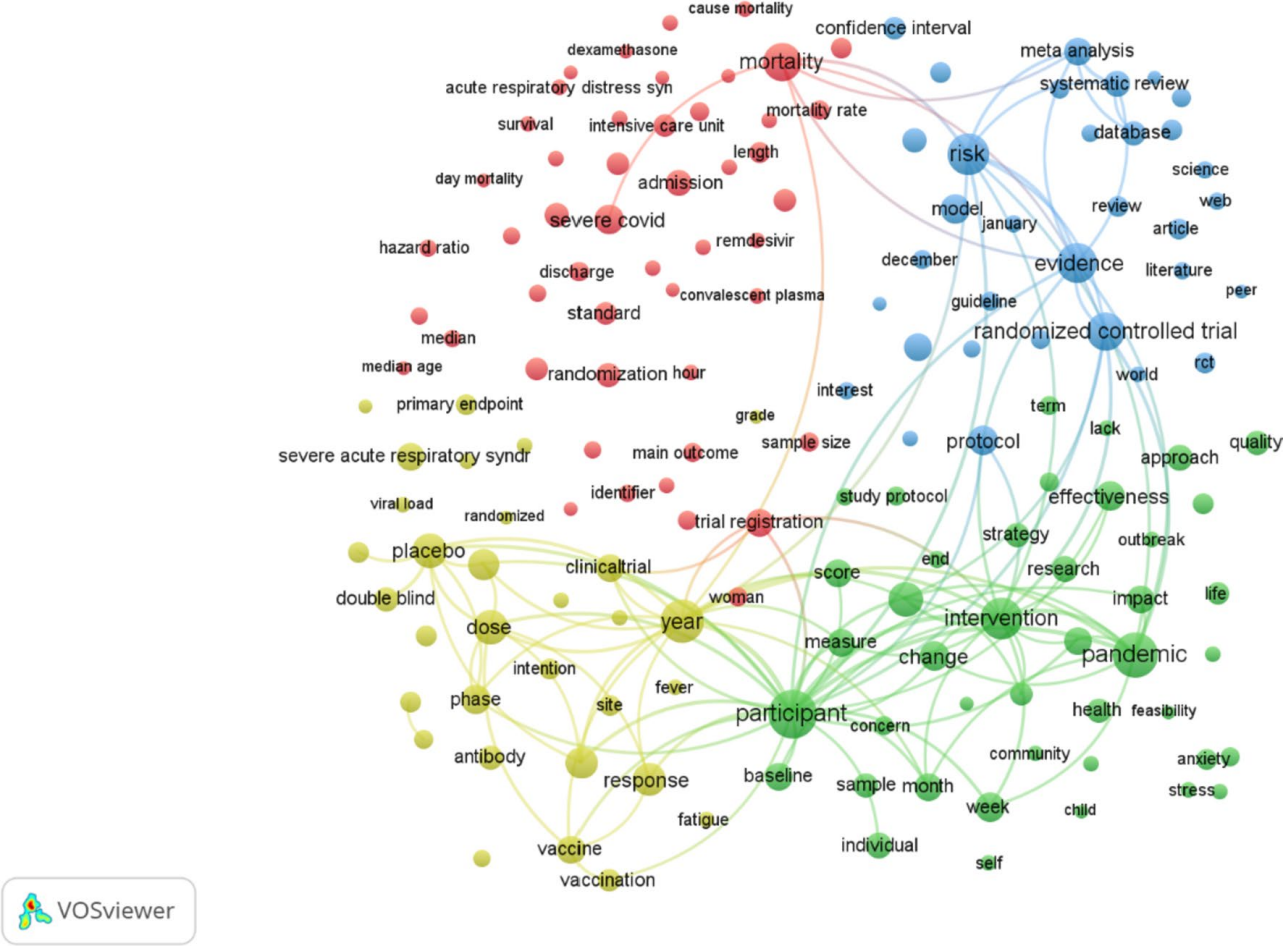
Cluster map based on term analysis appearing in titles or abstracts. The size of the circle indicates the occurrences of the terms, and the different colors indicate the variety of clusters. The map was created using VOSviewer software version 1.6.19

A co-occurrence network was created based on how often terms appeared in article titles and abstracts to identify the most important research topics in the field of COVID-19 RCTs. Figure 2 shows the most common research topics in the field of COVID-19 RCTs in the past three years. VOSviewer analysis was used to search for terms in the titles and abstracts of 3,727 documents. The map was created with 259 terms out of 664,923 terms, which were categorized into four clusters with at least 100 appearances per term. The most common terms were related to (a) the safety and effectiveness of COVID-19 vaccines [78, 84, 85, 88–94] (yellow cluster), (b) mental health strategies to cope with the impact of the pandemic [95–99] (green cluster), (c) the use of monoclonal antibodies to treat COVID-19 patients [100–105] (red cluster), and (d) systematic reviews and meta-analyses of COVID-19 research [72, 106–114] (blue cluster). This approach helped us pinpoint the most pressing issues in COVID-19 research and monitor the field's growth. You can see the clusters in Fig. 2.

### Future research direction analysis

**Fig. 3:**
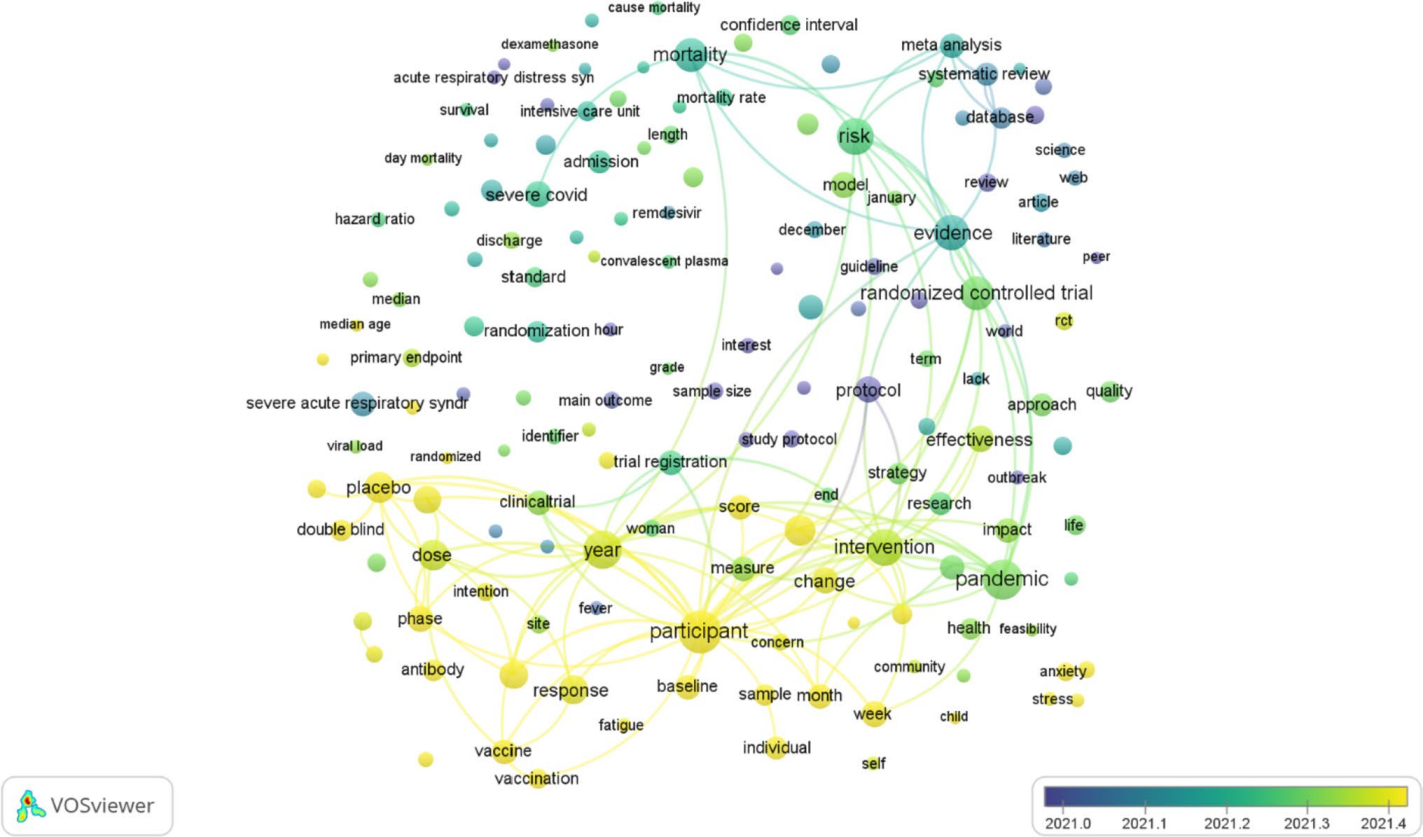
Network visualization map of the analysis of terms in titles and abstracts according to the frequency of appearance. Blue denotes earlier occurrences of the terms, and yellow denotes later occurrences. The map was created using VOSviewer software version 1.6.19

Figure 3 in VOSviewer employs a distinctive color scheme that assigns a unique color to each term based on its average frequency in all the publications that were retrieved. The color scheme follows a pattern where yellow denotes the most recent occurrences, while blue signifies the earliest occurrences. Prior to 2021, the field primarily focused on examining the connection between the use of neutralizing monoclonal antibodies and the risk of hospital admission and mortality in patients with COVID-19 and conducting systematic reviews and meta-analyses of randomized trials related to COVID-19. However, research on the "safety and immunogenicity of COVID-19 vaccines" and "mental health strategies to address the impact of COVID-19 on individuals' mental health" emerged more recently at the end of 2021 and beyond.

## Discussion

The descriptive findings of the current study shed light on COVID-19-related RCTs. The aforementioned data shed light on numerous publications, prestigious journals, research topics, and collaborative networks. The current findings shed light on the primary research foci and directions for COVID-19. These insights can help researchers, policymakers, and funders make informed decisions and identify research gaps. In the current COVID-19 pandemic, due to the unprecedented global death toll [115], scientific research has a decisive impact on the prevention and control of the disease. Despite the relatively short period in which much of the scientific literature on this topic has been generated, an investigation of pandemics is essential. This bibliometric analysis is notable for being the first effort to recognize and evaluate the features of the scientific literature on randomized controlled trials and COVID-19 that emerged during the early phases of the pandemic. The examination is established on a sample of 3,727 scientific papers that were published from January 2020 to December 2022. The sample delineates the contributions of different countries and institutions to the publications of COVID-19 RCTs. Furthermore, the study highlights the journals that published the most significant number of RCTs in the domain of COVID-19 research. Additionally, the analysis summarizes frequently cited publications and the primary research topics explored in this area.

Based on bibliometric analysis, the current results highlight the top countries and institutions in the main list. The base list refers to a series of journals widely recognized as the most prestigious and influential in this area. The current study reveals research productivity and country and institution influence. The top core list countries were high-income destination countries, including the US, UK, and Canada. It is not surprising that high-income countries rank high in bibliometric research studies because they have a strong research infrastructure, including funding, science and technology, and human resources [65–67, 116, 117]. Therefore, researchers in these countries benefit from having access to the newest technologies and research instruments to create high-quality results. In addition, the statistics demonstrate that *Harvard Medical School*, *Oxford University*, *Imperial College London*, and *Toronto University* are among the most active core list institutions in high-income countries. This reflects an outstanding research culture and motivated staff at these organizations. It also shows that these institutions' researchers have cutting-edge research facilities and resources.

The current investigation delineated four prominent research themes in RCTs and COVID-19 research by systematically analyzing terms and fields of research interest. The findings of this study revealed the most common terms in the scholarly literature and their prevalence in various publications. In particular, the theme of the safety and effectiveness of COVID-19 vaccines emerged as a prominent area of interest within current research. The current scientific consensus on the safety and effectiveness of COVID-19 vaccines has been established through extensive and rigorous clinical trials that show that these vaccines are highly effective in preventing infection with the SARS-CoV-2 virus and its associated disease, COVID-19 [118, 119]. Many large-scale studies in different populations have confirmed that COVID-19 vaccines have a high degree of safety with a minimum risk of serious side effects [119, 120]. Furthermore, real-life data from vaccine deployments in many countries provide convincing evidence that vaccines can significantly reduce hospitalization, serious illness, and mortality rates associated with COVID-19 [84, 121, 122].

Mental health strategies to address the impact of the pandemic have been a highly discussed topic during the COVID-19 outbreak. The COVID-19 pandemic has profoundly impacted global mental health [123]. The disturbances caused by the pandemic have led to a widespread epidemic of anxiety, depression, and other mental health problems [124]. Mental health strategy tests have become important to address these challenges [125, 126]. Mental health strategies focus on evaluating interventions that aim to reduce the impact of the epidemic on mental health. These interventions include psychological interventions such as cognitive behavior therapy, online self-help interventions, and telepsychiatry [127, 128]. In addition, non-pharmacological interventions such as physical exercise and social support have also been studied [129]. The pandemic has affected different populations in different ways, and testing mental health strategies must take into account these differences. For example, first-line healthcare workers were disproportionately affected by the pandemic, and mental health strategy trials targeting this population were launched [130, 131].

Another hot topic is ‘the use of monoclonal antibodies to treat COVID-19 patients’. Although some vaccines have been developed and approved for emergency use, the emergence of new variants and the slow onset of vaccination in some regions have highlighted the need for effective treatment options [15]. One promising approach is the use of monoclonal antibodies to treat patients with COVID-19 [132]. Monoclonal antibodies are laboratory proteins that mimic the immune system's ability to fight harmful pathogen viruses such as severe acute respiratory syndrome coronavirus-2 [133]. They are specifically designed to target a specific antigen on the surface of a virus, in this case, the SARS-CoV-2 spike protein [134]. Several monoclonal antibodies against SARS-CoV-2 have been developed, including bamlanivimab, casirivimab, and imdevimab, which have received emergency approval from the US Food and Drug Administration [135, 136]. Clinical studies have revealed that monoclonal antibodies can reduce the risk of hospitalization and death in patients with COVID-19 who are not hospitalized and are at high risk of developing severe disease [137, 138].

Another current hot issue is “the systematic reviews and meta-analyses of RCTs related to COVID-19 research”. Systematic reviews and meta-analyses are frequent in medical research. Due to the rapid progress and amount of research productivity, these approaches are ideal for COVID-19 study. Systematic reviews and meta-analyses enhance clinical decision-making and public health policy by summarizing relevant research [139, 140]. Recent data indicate that most COVID-19 systematic reviews and meta-analyses are conducted in countries with high incomes, including the US, UK, and Canada [140]. This is expected since these countries have research infrastructures, funds, equipment, and qualified investigators [141, 142]. However, it should be noted that COVID-19 research concentrated on research from high-income countries could also be considered a limitation. This is because COVID-19 affects countries all over the world, including many low- and middle-income countries where resources and research infrastructure may be limited [143]. Therefore, researchers and policy makers must ensure that the results of COVID-19 research conducted in high-income countries are relevant to the global context [144, 145].

Based on the topic maps analyzed, it is evident that a significant number of scholarly works have been published that focus on two central themes related to the COVID-19 pandemic: the safety and efficacy of COVID-19 vaccines and mental health strategies aimed at mitigating the adverse impact of the pandemic on individuals' psychological well-being. In particular, these two themes have gained prominence in recent years; however, the COVID-19 outbreak has heightened their significance and urgency. Given the sustained effects of the pandemic and the persistent need for mental health support, it is probable that these topics will continue to be of paramount importance in the foreseeable future. Consequently, additional research efforts and focused attention are imperative to effectively address the novel challenges in these fields. Urgency, public interest, existing expertise, innovation potential, and collaboration all influence the prominence of research fields [146, 147]. Priority is often given to research fields that have the potential to offer innovative solutions to prevalent issues. Finding effective treatments and preventive measures is crucial in the case of COVID-19. The study shows that COVID-19 vaccines, mental health strategies, monoclonal antibodies, and systematic reviews were the most prevalent research topics. These areas directly address crucial pandemic aspects, such as vaccine development, mental health support, and therapeutic interventions [148–151]. In addition, research fields that foster collaboration and networking among researchers tend to expand more rapidly. Collaborative efforts can result in interdisciplinary insights and broader research outcomes. The study's co-occurrence analysis identified four clusters of COVID-19-related research areas. These clusters presumably represent collaborative networks of researchers working on specific topics, indicating that collaboration has shaped the research landscape [64, 152–154].

## Future and clinical implications of the current study

By highlighting the disparities and considering the progression of research throughout this pandemic, potential implications for worldwide health research and application can be indicated as follows:

1. The study covers the most common COVID-19 RCT themes, including vaccinations, mental health, monoclonal antibodies, and systematic reviews. Data can help researchers, policymakers, and funders focus on the most important locations of pandemics.
2. Policymakers and funders can strategically allocate resources using this study. Identifying research groups such as mental health methods and monoclonal antibody treatments helps invest in pandemic research that could improve clinical results and public health.
3. Anticipated research directions recognize emerging research disciplines. Policymakers and funding organizations can use this evidence to anticipate trends and allocate resources to support research on COVID-19 and other challenges and opportunities.
4. The method can be used to identify research gaps in future pandemics and global health emergencies. By evaluating the strengths and shortcomings of the COVID-19 research response, the global health community can better manage and mitigate these outbreaks.

## Strengths and limitations

This study represents the most comprehensive and up-to-date bibliometric analysis of COVID-19 RCTs available, providing valuable information for patients, therapists, and researchers. However, it is important to acknowledge several limitations. First, the study used the Scopus database exclusively to identify relevant articles, which is widely considered a reliable and comprehensive source in many academic fields but may have resulted in the omission of publications from other databases, such as PubMed and Web of Science [155–159]. Second, the study used a comprehensive list of keywords derived from previous literature reviews [59–62, 69–72], yet it is possible that some relevant keywords were overlooked, potentially leading to false negative results. Third, the study selected the ten most frequently cited articles, but the citation count is time dependent, and older papers are more likely to be cited, potentially biasing the selection of highly cited articles. Fourth, the study limited the search to terms related to COVID-19 RCTs in the title and abstract only, which may have excluded relevant articles that used these terms elsewhere in the text. Finally, the use of Scopus data may not fully capture the research output of active institutions with multiple Scopus profiles or funding agencies identified by various names in published papers. Therefore, to minimize bias, it is essential to restrict data analysis related to the most active institutions and funding agencies to the results obtained from Scopus without any manipulation or merging.

## Conclusions

This bibliometric analysis revealed that the research evidence available in recent years, especially last year, has steadily increased. The results conclude that the research on COVID-19 RCTs has been a collaborative effort between countries, institutions, and funding institutions. The United States, the United Kingdom, China, and Canada contributed the most to the publications. *Harvard Medical School, Oxford University*, and *Imperial College London* are the leading universities actively involved in the production of research articles. The *National Institutes of Health*, *China's National Natural Sciences Foundation,* and the *National Institutes of Allergy and Infectious Diseases* provide the highest funding for research articles. *Trials, BMJ Open, Plos One*, and *JAMA Network Open* are the most active journals that publish articles on COVID-19 RCTs. Co-occurrence analyses identified four main research areas: vaccine safety and effectiveness, mental health strategies, monoclonal antibody treatment, and systematic review and meta-analysis of COVID-19 RCTs. It is recommended that future research on COVID-19 RCTs continue to focus on these four areas. Collaborating with countries, institutions, and funding bodies should encourage support and funding for research in these fields. The most active journals should continue to publish research on these subjects. Continued research in these areas will provide valuable information contributing to global efforts to control and manage the COVID-19 epidemic. In the global health field, this study's implications highlight the importance of strategic research planning, collaboration, and data-driven decision-making. By acknowledging the achievements and gaps, the global health community can work more effectively to combat the ongoing pandemic and establish a solid foundation to address future health challenges.

## Data Availability

All data generated or analyzed during this study are included in this published article. In addition, other datasets used during the current study are available from the author on reasonable request (saedzyoud@yahoo.com).

## Abbreviations

COVID-19: Coronavirus disease 2019
RCTs: Randomized controlled trials
FDA: Food and Drug Administration
WHO: World Health Organization
MeSH: Medical Subject Headings
